# Time estimation and arousal responses in dopa-responsive dystonia

**DOI:** 10.1038/s41598-022-17545-w

**Published:** 2022-08-22

**Authors:** Leonie F. Becker, Sinem Tunc, Peter Murphy, Tobias Bäumer, Anne Weissbach, Martje G. Pauly, Duha M. Al-Shorafat, Gerard Saranza, Anthony E. Lang, Christian Beste, Tobias H. Donner, Julius Verrel, Alexander Münchau

**Affiliations:** 1 Institute of Systems Motor Science, Center of Brain, Behavior and Metabolism, Universität zu Lübeck, Ratzeburger Allee 160, 23538 Lübeck, Germany; 2 Department of Neurology, University Hospital Schleswig Holstein, Lübeck, Germany; 3 Section Computational Cognitive Neuroscience, Department of Neurophysiology and Pathophysiology, University Medical Center Hamburg-Eppendorf, Hamburg, Germany; 4 School of Psychology, Trinity College Institute of Neuroscience, Trinity College Dublin, Dublin, Ireland; 5 Bernstein Center for Computational Neuroscience Berlin, Berlin, Germany; 6 Institute of Neurogenetics, Universität zu Lübeck, Lübeck, Germany; 7 Edmond J. Safra Program in Parkinson’s Disease, Morton and Gloria Shulman Movement Disorders Clinic, Toronto Western Hospital, Toronto, ON Canada; 8 Neuroscience Department, Jordan University of Science and Technology, Irbid, Jordan; 9 Section of Neurology, Department of Medicine, Chong Hua Hospital, Cebu City, Philippines; 10 Cognitive Neurophysiology, Department of Child and Adolescent Psychiatry, Faculty of Medicine, TU Dresden, Dresden, Germany

**Keywords:** Cognitive neuroscience, Motor control, Neurological disorders, Dystonia, Movement disorders

## Abstract

Dopa-responsive dystonia (DRD) is caused by an impaired dopamine biosynthesis due to a GTP-cyclohydrolase-1 (GCH1) deficiency, resulting in a combination of dystonia and parkinsonism. However, the effect of *GCH1* mutations and levodopa treatment on motor control beyond simple movements, such as timing, action preparation and feedback processing, have not been investigated so far. In an active time estimation task with trial-by-trial feedback, participants indicated a target interval (1200 ms) by a motor response. We compared 12 patients tested (in fixed order) under their current levodopa medication ("ON") and after levodopa withdrawal ("OFF") to matched healthy controls (HC), measured twice to control for repetition effects. We assessed time estimation accuracy, trial-to-trial adjustment, as well as task- and feedback-related pupil-linked arousal responses. Patients showed comparable time estimation accuracy ON medication as HC but reduced performance OFF medication. Task-related pupil responses showed the reverse pattern. Trial-to-trial adjustments of response times were reduced in DRD, particularly OFF medication. Our results indicate differential alterations of time estimation accuracy and task-related arousal dynamics in DRD patients as a function of dopaminergic medication state. A medication-independent alteration of task repetition effects in DRD cannot be ruled out with certainty but is discussed as less likely.

## Introduction

Dopa-responsive dystonia (DRD) is caused by mutations in the *GCH1* gene impairing the function of GTP-cyclohydrolase-1, the rate-limiting step of dopamine synthesis in the brain, leading to deficits of dopamine in the brain^1^. Clinically, it presents with dystonia and parkinsonism in various combinations^2^. DRD is considered a neurometabolic disorder associated with little or no neurodegeneration^3,4^. Dopamine deficiency impairs the function of nigrostriatal neurons, which project to striatal D1 receptors predominantly on medium spiny neurons (MSN) in the striosomal compartment, affecting the direct pathway of the basal ganglia^4,5^. In addition, DRD has also been associated with abnormally increased activity in the dorsal midbrain, the cerebellar vermis, and the supplementary motor area (SMA)^6^. These and other findings differ from patients with Parkinson’s disease (IPD). For example, in patients with IPD, neurodegeneration not only affects the dopaminergic but also the noradrenergic neurotransmitter system with pronounced and early involvement of the locus coeruleus^7–9^.

While motor symptoms^1,2,4,5^ are relatively well-defined in DRD, much less is known about dysfunctions of motor control beyond simple movements including timed motor responses and arousal responses. The aim of our study was to expand knowledge of this rare disease, focusing on implications of the levodopa treatment, which is interesting for medical practitioners covering patients with DRD but also other diseases with dopamine deficits.

Interval timing appears as a particularly suitable task to be examined in DRD because it involves cortico-striatal circuits including MSNs and the SMA, as well as the cerebellum and is modulated by the dopaminergic system^10–15^. According to the Scalar Expectancy Theory^10^, interval timing is mediated by a pulse-generating pacemaker. MSNs in the dorsal striatum are thought to balance incoming oscillatory cortical and thalamic signals functioning as a memory and decision stage^10^. Dopaminergic release from the ventral tegmental area (VTA) acts as a synchronization signal for cortical oscillatory cells. Release from the substantia nigra works as a reset signal for MSNs at the beginning of a time estimation. Output from the striatum then travels via direct and indirect pathways, thalamus and again cortex and striatum, influencing the neuronal oscillatory rate and therefore clock speed^10^. Different concentrations of tonic dopamine in the brain can lead to increasing or slowing the rate of the internal pacemaker^13^, resulting in underestimation of time with higher dopamine levels and overestimation of time with lower dopamine levels^13^. Furthermore, D1 dopamine receptor blocking leads to less motivation to respond in a time estimation task, with more prominent results with stronger receptor blocking^16^. Against this background, we predicted withdrawal of dopaminergic medication (“OFF”) to result in degraded time estimation accuracy in DRD patients, with a more pronounced performance degradation in patients with higher medication levels, in whom withdrawal may lead to a more pronounced deficiency.

In addition, because dopamine is the precursor of noradrenaline, dopamine deficiency in DRD patients is expected to also influence functions mediated by the noradrenergic system. Noradrenaline concentration has been shown to be reduced in the blood of DRD patients^17^ and the concentration of the noradrenaline metabolite 3-methoxy-4-hydroxyphenylglycol is reduced in the cerebrospinal fluid^18^. The activity of the noradrenergic locus coeruleus affects arousal, attention and motivation^19,20^, selectively promoting task-relevant behaviors engaged by task-related decision processes^19^.

In the present study, functioning of the temporal attentional filter may be important for time estimation, because the timing system requires attention^12^. Pupil dilation as a marker of brainstem arousal systems^21^ can be used to study the integrity of this system. Apart from changes in noradrenergic concentrations, the dopaminergic system could also influence the pupil-linked arousal system because the former is closely connected to the latter, both anatomically^22^ and functionally^21^. Recent studies demonstrate the importance of the dopaminergic system for the control of pupil-linked arousal systems^21,23^. In particular, pupil dilatation, as a reflection of brainstem arousal systems^21,24,25^, has been observed after performance-related feedback^23^, scaling with uncertainty about the preceding decision^26^. In addition, tasked evoked pupil responses have an influence on decision bias and correlate with the activity in the ventral tegmental area (VTA)^21^. This shows analogies to dopaminergic activity during an ambiguous decision task in monkeys^27^. Consequently, we expected that task-related arousal dynamics would parallel altered time estimation performance in these patients, including potential associations with medication levels. On the other hand, levodopa doses are predominantly titrated as a function of motor symptoms, so that basal ganglia would get the right amount of dopamine. Other areas with different dopamine sensitivity, like the VTA, may be overdosed. In ON state, this could lead to a distorted pupil reaction.

## Methods

### Participants and clinical assessment

Data from twelve patients (43.58 ± 18.21 years; 11 females) and twelve healthy controls (HC; 43.42 ± 18.22 years; 11 females) were included in our study. Patients had genetically confirmed DRD with mutations in the *GCH1* gene and were treated with levodopa, levodopa with a DOPA decarboxylase inhibitor and dopamine agonists (cabergoline, rotigotine). In addition, two patients took antidepressants (citalopram, mirtazapine) and one trihexyphenidyl. To standardize the intake of dopaminergic drugs, we calculated the levodopa equivalence dose per participant per day and calculated the body-weight adjusted levodopa intake^28,29^. The average daily dose was 275 mg (SD 212 mg, range 25 mg-700 mg), the corresponding body weight adjusted dose was 4.22 mg/kg (SD 3.62 mg/kg, range 0.36–11.29 mg/kg). Patients were investigated twice, first under their regular dopaminergic medication (ON) and then after medication withdrawal (OFF), on average 23.2 h (± 7.1 h) after their last medication intake.

Sessions were not counterbalanced for two main reasons. First, most patients travelled longer distances for taking part in the study and being off medication would have made the trip too troublesome for many of them. Second, and more importantly, while addressing basic repetition effects (e.g., between-session learning) as a potential confound, counterbalancing would likely have introduced new confounds. For instance, dopaminergic state when taking medication again after a one-day withdrawal (when counterbalancing order) may not be equivalent to the steady state after continuous medication (as in our current design). In addition, given the close connection between dopamine status and learning^30–34^, medication withdrawal may not only affect current performance but also performance in subsequent sessions. Studying half of the patients OFF medication in the first session would very likely introduce an additional Group × Medication × Session Order interaction. Due to the limited number of participants, it would not be possible to disentangle these confounding factors from the main experimental manipulation. Instead, counterbalancing would add unexplained variability to the outcome measures, thereby increasing measurement noise and making the detection of experimental effects less likely. We acknowledge that the decision not to counterbalance results in a confound between medication and session order interpretation of results (see “Discussion”).

With two exceptions, the two sessions took place on consecutive days. HC were individually matched to patients by gender, age (± 5 years) and handedness. None of them were diagnosed with a psychiatric or neurological disorder. All HC were measured twice on consecutive days to control for repetition effects (Session 1/Session 2). To minimize the influence of other sources of variability, patients and healthy controls were measured approximately at the same time of the day for the first and second measurement.

Patients were characterized clinically by video rating, blinded regarding medication state, using part III (motor) of the International Parkinson and Movement Disorder society revised Unified Parkinson’s Disease Rating Scale (MDS-UPDRS)^35^ and the Burke-Fahn-Marsden dystonia rating scale (BFMDRS)^36^. Both patients and HC performed a nine-hole peg test (NHPT)^37^. All participants provided written informed consent. The study was approved by the ethics committee of the Universität zu Lübeck and performed in accordance with the Declaration of Helsinki.

### Experimental setup and procedure

**Figure 1:**
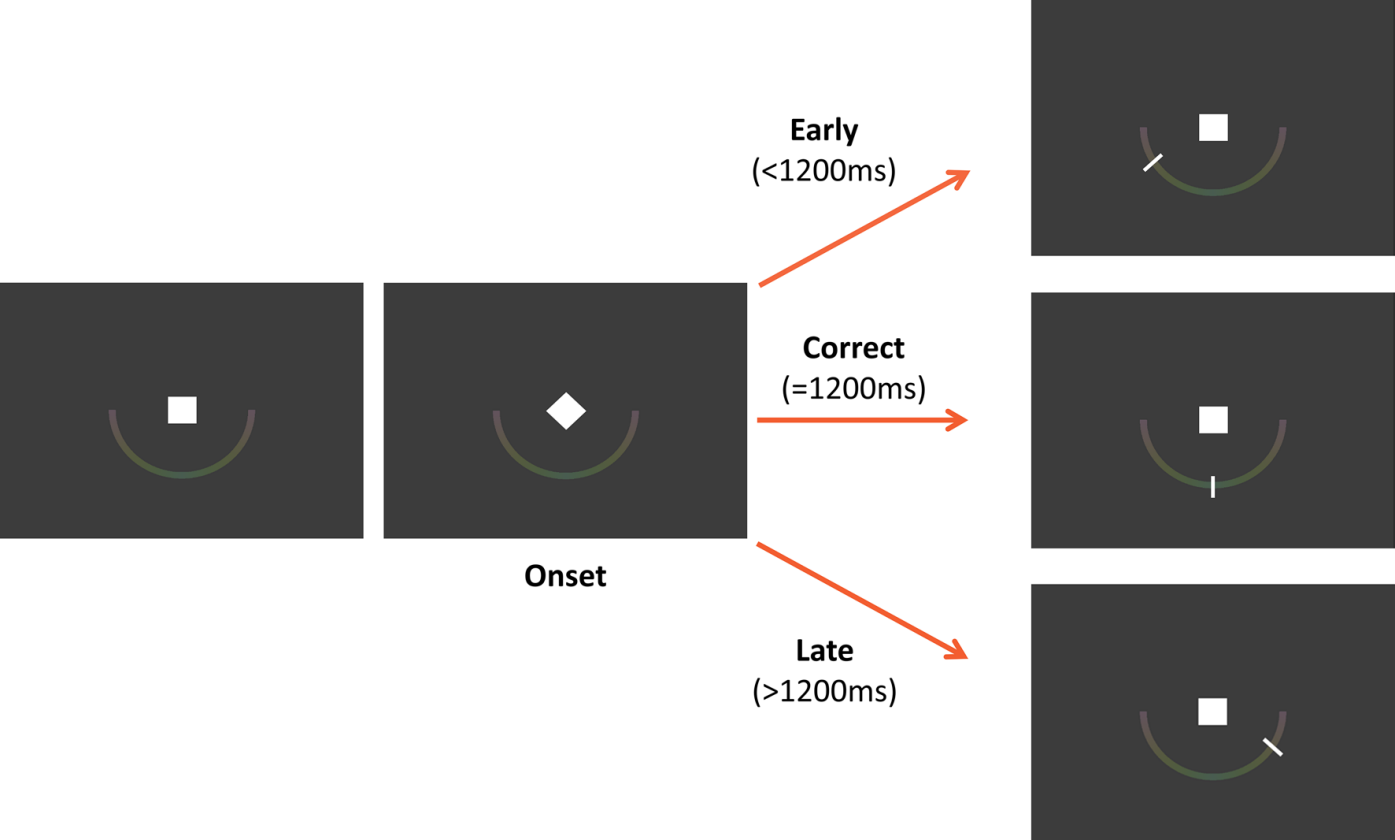
*Time estimation (TE) task.* Participants were asked to press a button 1200 ms after a start signal (rotation of a white square), with feedback on performance accuracy given on a continuous scale immediately after the button press.

Participants were seated in front of a computer screen in a darkened room. The time estimation task was implemented in Matlab (Version 9.1 (R2016b)), using the PsychToolbox 3^38,39^. A white square (25 mm x 25 mm) and a light, approximately isoluminant color scale (light red–light green–light red) with the shape of a lower semicircle were continuously shown on screen on a black background (Fig. 1). At trial onset, the square was rotated by 45°. Participants were instructed to press the response key with their dominant hand as close to 1200 ms after trial onset as possible. The interval of 1200 ms was chosen, as it has previously been used in patient groups with different dopaminergic involvements or basal ganglia disorders^11,40,41^. Immediately after the response, participants received visual feedback by a white marker positioned on the color scale. For an accurate response (1200 ms), the marker would appear at the lower-middle position on the semicircle. For shorter or longer intervals, the marker appeared on the left or right. Intervals outside the range from 600 to 1800 ms were mapped to the boundaries. Trials automatically ended after 2000 ms. The task had nearly the same luminance throughout the experiment.

After task familiarization and practice trials, the main experiment consisted of 120 trials, presented in two blocks. The intertrial interval was jittered between 1700 and 2300 ms.

Behavioral data were analyzed using custom-made Matlab scripts. The mean response time and the mean absolute deviation ($|error$|) from the target interval (1200 ms) were determined per participant and session. Trial-to trial adjustments of response times were assessed through single-trial regression, defining an “adjustment score” quantifying to what extent participants sped up following an especially slow response, and slowed down following an especially fast response. To do so, we fitted the following regression model:

$$\Delta R{T}_{t}={\beta }_{0}+{\beta }_{1}\cdot R{T}_{t-1}+{\beta }_{2}\cdot R{T}_{t-1}\cdot |error{|}_{t-1}$$

where $R{T}_{t-1}$ denotes the RT on trial *t* − 1, $|error{|}_{t-1}$ denotes the absolute error on trial *t* − 1, and $\Delta R{T}_{t}$ denotes the change in RT from trial *t* − 1 to trial *t* (i.e. $R{T}_{t}-R{T}_{t-1}$, yielding positive values for a slowing down of RT on trial *t* and negative values for a speeding up). The rationale behind this model is that ${\beta }_{1}$ will capture the change in RT across adjacent trials which is due to regression to the mean expected to yield a strong negative coefficient for ${\beta }_{1}$. ${\beta }_{2}$, on the other hand, will capture the change in RT *over and above* regression to the mean that is specifically driven by the magnitude of the error on the previous trial. Thus, a negative "adjustment coefficient" ${\beta }_{2}$ would indicate that participants sped up following an especially slow response and slowed down following an especially fast response.

### Pupil response

Pupil data were acquired at 60 Hz using a binocular head-mounted eye-tracking system (Pupil Labs GmbH, Berlin, Germany) with manufacturer-supplied software (Pupil Capture, Version 1.8.26). For each participant and session, we selected the pupil diameter time series (left/right eye) with the larger proportion of high-quality samples, based on a confidence rating assigned to each sample by the pupil tracking software and a manufacturer-recommended threshold of 0.6. We then linearly interpolated over artifacts identified via the confidence score (same threshold of 0.6) and a threshold on the first derivative of the pupil signal (4 SD), as described in Murphy et al.^42^ and implemented via code shared here: https://github.com/DonnerLab/2021_Murphy_Adaptive-Circuit-Dynamics-Across-Human-Cortex/. The resulting time-series was high-pass filtered to 0.06 Hz and low-pass filtered to 6 Hz (Butterworth, 3rd order) to remove slow drifts in the pupil outside of the trial-evoked dilations and non-physiological noise. Finally, the time series was normalized to percentage of the median pupil size (determined per block and prior to filtering).

The cleaned, filtered, and normalized pupil signal was segmented relative to the time of response (identical to the time of feedback), using two different time windows and baseline corrections to address task-related and feedback-related pupil effects, respectively. For the task-related analysis, the time segmentation window was [−1500 ms, 1000 ms] *relative to the response*, baseline-corrected by subtracting the per-trial average in the 400 ms preceding the *stimulus*. Based on known cognitive-pupil delays of 500–1000 ms^43^, we expected the task-related pupil response to peak up to 1000 ms after the response. The prolonged period of 1500 ms preceding the response was included in the segmentation interval to illustrate the build-up of the pupil response from trial onset to response. Baseline pupil size was determined by a separate segmentation ([−400 ms, 0] relative to stimulus), as the baseline interval was usually not contained in the segmentation interval (and the overlap varied due to response time variability). For feedback-related effects, the analysis time window was [0, 1500 ms] and the baseline window was [−400 ms, 0], both relative to the time of the response/feedback. Trials with a response time below 400 ms as well as any segment in which > 50% of samples were artefactual or that overlapped with an artefactual period that was longer than 750 ms were discarded from subsequent analyses. The remaining segments were averaged per participant and session, to determine the average individual pupil response.

Task-related effects were investigated in terms of the mean response-locked change in pupil size. We hypothesized that the pupil would show a task-related dilation relative to the pre-stimulus baseline. This was assessed in a time-resolved analysis, comparing for each time point of the task-related segment the average of both sessions for all participants (across both groups) to 0. For a univariate analysis of Group and Session effects, we extracted the average pupil dilation in a time window of ± 250 ms around the pupil dilation peak (determined across participants and sessions). Robustness was assessed by repeating the analysis with a smaller time window (± 50 ms).

Feedback-related effects on pupil dilation were assessed based on the response-/feedback-locked average segments with response-locked baseline correction. The sensitivity of the pupil response to performance ($|error|$, i.e., absolute deviation from the target interval 1200 ms) was assessed by a time-resolved linear regression of single-trial pupil dilation on $|error|$. That is, for each time point of the feedback-locked analysis interval, we determined the coefficient ${\beta }_{1}$ in the linear regression $d \sim { \beta }_{0}+ {\beta }_{1} \cdot |error|$ across trials, where $d$ denotes per-trial pupil dilation and $|error|$ denotes performance error. The mean feedback-related pupil response, as well as the regression coefficients were submitted to the same analysis as described above for task-related changes, however extracting univariate data in both cases based on the peak time determined from the regression analysis.

### Statistical analysis

Statistical analyses were performed in R (Version 4.1.0) and JASP (Version 0.11.1.0). Behavioral and pupillometric experimental measures per participant and session were analyzed with two-way ANOVAs with Group as between- and Session as within-subject factor.

For the sequential adjustment score, we assessed whether it significantly deviated from 0 (using one-sample t-tests per Session and Group).

MDS-UPDRS-III scores, BFMDRS scores and time needed for the NHPT were compared in DRD patients between ON and OFF state by Wilcoxon signed-rank tests. NHPT results were additionally contrasted between DRD patients and HC via Mann–Whitney tests, separately for the first and second measurements (ON/Session 1, OFF/Session 2).

In DRD patients, behavioral performance (mean absolute error) and pupil responses were correlated with MDS-UPDRS-III score, BFMDRS score and NHPT, separately for ON and OFF states, as well as in terms of between-session changes (i.e., OFF minus ON, both for experimental and clinical measures). Between session changes in performance and pupil response (OFF minus ON) were correlated to the daily dosage of levodopa, normalized to body weight. These correlations are reported as exploratory analyses, not corrected for multiple comparisons.

## Results

### Clinical results

DRD patients motor impairments were more pronounced in OFF compared to ON state according to MDS-UPDRS-III (W = 1, p = 0.005, ON: 6.22 ± 6.66, OFF: 10.33 ± 9.85) and BFMDRS (W = 11, p = 0.027; ON: 9.93 ± 9.38, OFF: 13.54 ± 13.72). In the NHPT, DRD patients showed significantly lower performance compared to HC both ON (both hands: W = 112, p = 0.020; DRD: 23.02 ± 4.27, HC: 19.39 ± 1.92; dominant hand: W = 115, p = 0.012; DRD: 21.95 ± 3.88, HC: 18.73 ± 2.17) and OFF medication (both hands: W = 122, p = 0.003; DRD: 22.89 ± 4.72, HC: 18.85 ± 1.13; dominant hand: W = 114, p = 0.014; DRD: 21.36 ± 3.47, HC: 18.33 ± 1.44). Medication did not have a significant effect on NHPT performance in patients (p = 0.3).

### Behavioral performance

**Figure 2:**
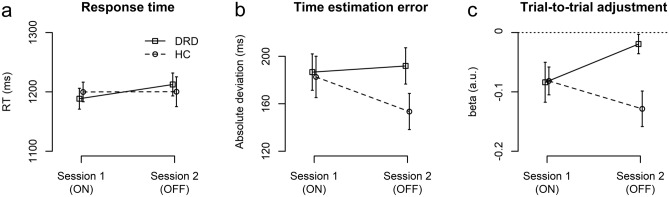
*Behavioral performance.* Response times (**a**), magnitude of time estimation errors (**b**), and trial-to-trial adjustment (**c**) (regression coefficient ${\beta }_{1}$ with more negative values indicating stronger adjustment) for the two sessions and subject groups. Error bars indicate standard error of the mean.

Results from the behavioral analysis are shown in Fig. 2. The mean response time did not show any significant main or interaction effects of Group and Session (all p > 0.4). For the mean absolute error ($|error|$), the main effects were nonsignificant (p > 0.1) but a significant Group × Session interaction (F(1,22) = 4.56, p = 0.044, ${\eta }_{p}^{2}$=0.172) indicated differential changes from Session 1 to 2 in the two groups. As residuals deviated from normal distribution (Shapiro–Wilk test), we re-analyzed the Group × Session interaction using a non-parametric Mann–Whitney test, comparing the Session effect (Session 2 minus Session 1) between groups. This confirmed the presence of group-differential session effects (W = 112, p = 0.02). While both groups showed similar accuracy in Session 1 (ON), patients’ performance degraded relative to HC in Session 2 (OFF).

Analyses of the sequential adjustment scores indicated a main effect of Group (F(1,22) = 4.66, p = 0.042, $${\eta }_{p}^{2}$$=0.175) and a marginally non-significant Group × Session interaction (F(1,22) = 3.81, p = 0.063, $${\eta }_{p}^{2}$$=0.148). One-sample t-tests showed adjustment scores significantly deviated from zero in HC in both sessions (Session 1: $${p}_{adj}=0.016$$, Session 2: $${p}_{adj}=0.005$$), while in the DRD group this was only a trend in Session 1 ($${p}_{adj}=0.060)$$ and non-significant in Session 2 ($${p}_{adj}=0.26$$). More negative adjustment scores indicate stronger trial-to-trial adjustment in HC compared to DRD overall, and a trend towards differential effects from Session 1 to Session 2, suggesting a further reduction in trial-to-trial adjustment in DRD, which, moreover, did not significantly differ from 0 in Session 2.

### Pupil responses

**Figure 3:**
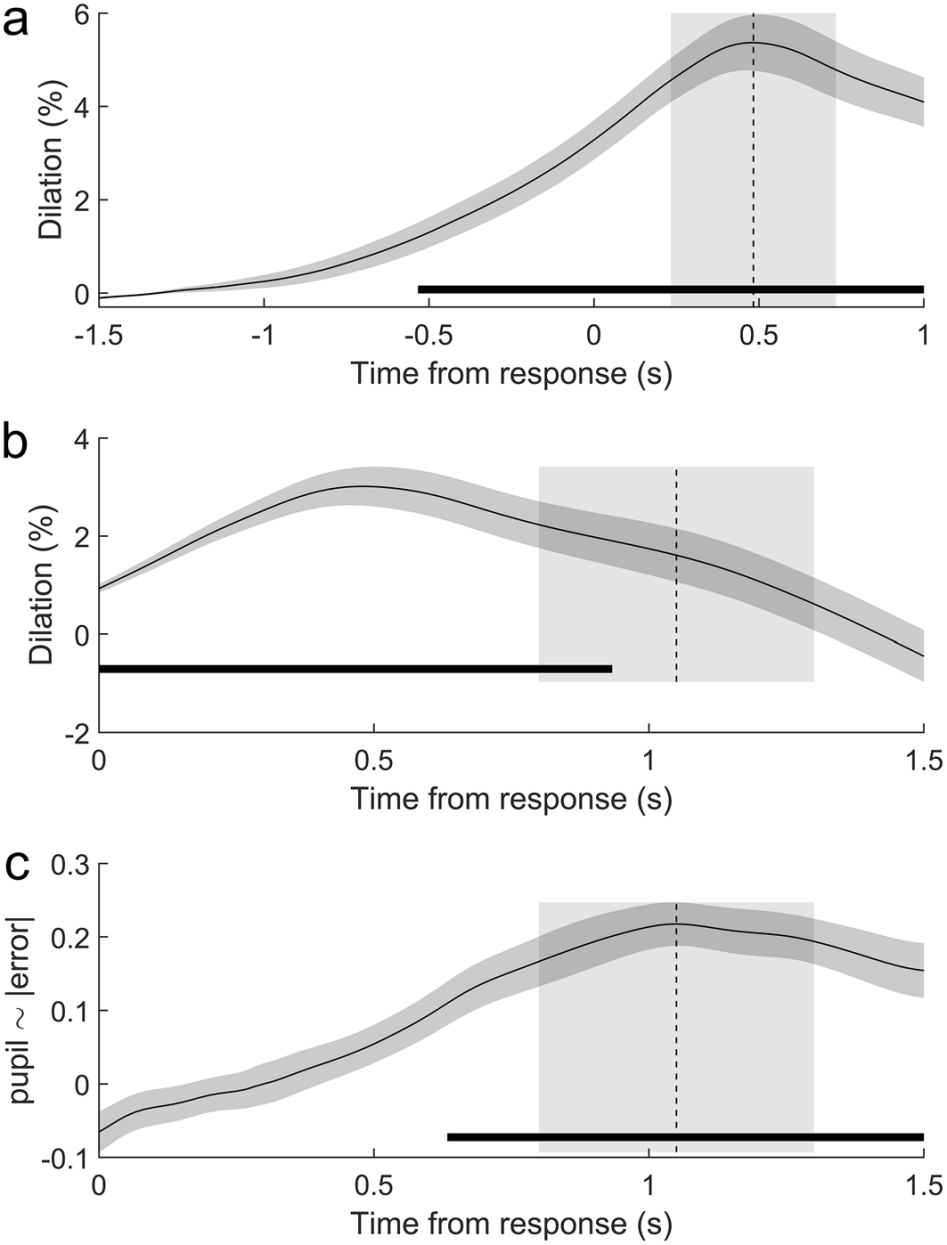
*Pupil traces during time estimation task.* Pupil traces (mean and standard error) baseline-corrected relative to a pre-stimulus (**a**) and pre-response period (**b**), as well as regression of feedback-related pupil-response on performance error (**c**). Data are time-locked to the time of response (and feedback).

Pupil diameter gradually ramped up during the trial in both groups and both sessions, peaking 0.483 s after the response (Fig. 3a). The time-resolved analysis revealed a significant task-related dilation in a time interval beginning 0.533 s before the response and extending until the end of the analysis interval, 1 s after the response.

**Figure 4:**
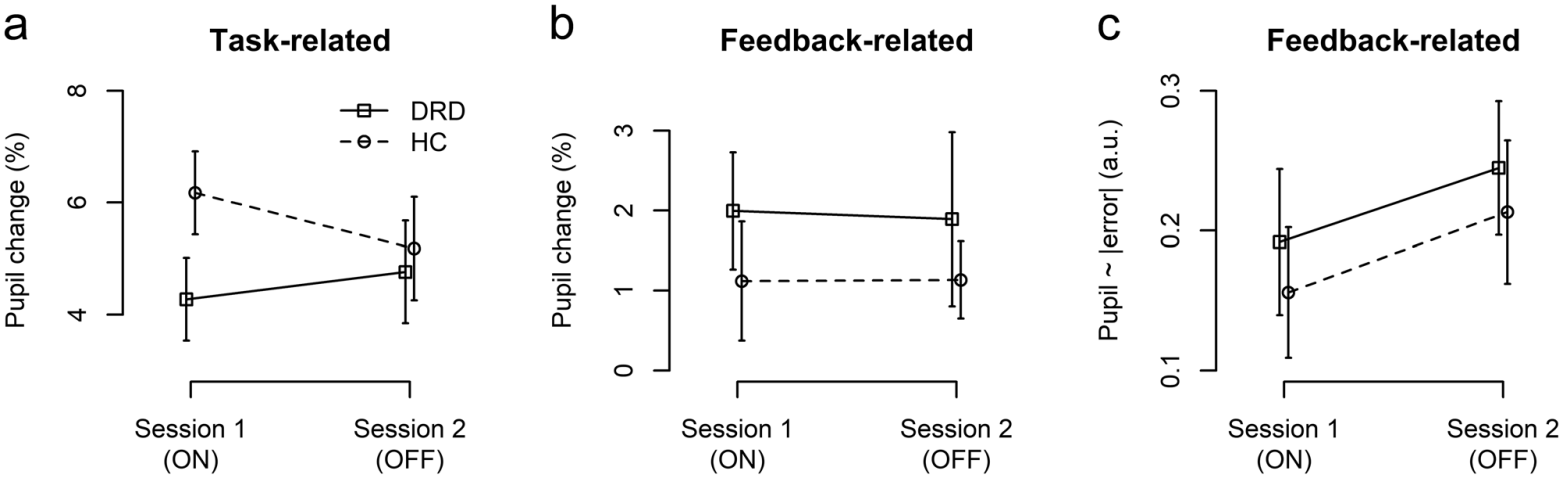
*Pupil dilation effects. *Task-related (**a**) and feedback-related change in pupil diameter (**b**) as well as regression of single-trial feedback-related pupil dilation onto $|\mathrm{error}|$ (**c**). Error bars indicate standard error of the mean.

In the Group x Session ANOVA (Fig. 4a), task-evoked pupil responses extracted ± 0.25 s around the pupil peak (0.483 s), showed a significant Group x Session interaction (F(1,22) = 5.25, p = 0.032, ${\eta }_{p}^{2}$=0.193) and no significant main effects of Group or Session (p > 0.3). Task-related pupil responses were (numerically) less pronounced in DRD patients compared to HC in Session 1 (ON), and the difference between the groups was reduced in Session 2 (OFF). The peak dilation determined using a smaller time window (± 50 ms) showed the same pattern (Group x Session: F(1,22) = 5.80, p = 0.025, ${\eta }_{p}^{2}$=0.018). An additional analysis using linear mixed effects models, statistically controlling for mean “raw” (non-normalized) pupil diameter and mean RT per participant and session^44^, confirmed these results.

The feedback-related pupil dilation showed a similar temporal pattern (Fig. 3b). The feedback-related pupil dilation extracted ± 0.25 s around the maximal regression coefficients (1.05 s, Fig. 4b), did not show any main or interaction effects of Group or Session (all p > 0.4). Regressing the single-trial feedback-related pupil dilation onto $|error|$ (Fig. 3c) showed a positive association between performance error (indicated by the visual feedback) and pupil dilation ramping up from about 0.5 s after response/feedback and peaking at 1.05 s. This is again consistent with a cognitive-pupil delay of 0.5–1 s^43^, now reflecting cognitive processing of the visual feedback. Indeed, the time-resolved analysis indicated significant (positive) deviation of regression coefficients from 0 in a time interval starting 0.633 s after response/feedback until the end of the analysis interval. Peak-extracted data (1.05 s ± 0.25 s, Fig. 4c) only showed a weak trend towards an effect of Session (F(1,22) = 3.08, p = 0.093) and no other main or interaction effects (p > 0.5).

### Correlation of clinical, behavioral and pupillometric data

The following explorative analyses are reported without correction for multiple comparisons and therefore need to be interpreted with caution.

Mean absolute error (|error|) in ON state showed a significant positive correlation with NHPT for the dominant hand (Pearson’s r = 0.603, p = 0.038) and trends for a correlation with NHPT for both hands (r = 0.555, p = 0.061) and MDS-UPDRS-III (r = 0.500, p = 0.098). In OFF state, a significant positive correlation with NHPT for the dominant hand was present (r = 0.676, p = 0.016), as well as trends for a correlation with NHPT for both hands (r = 0.523, p = 0.081) and MDS-UPDRS-III (r = 0.577, p = 0.050). However, the corresponding changes between sessions (i.e., difference scores OFF minus ON) did not indicate significant associations (p > 0.6; for BFMDRS and NHPT for both hands an extreme outlier had to be excluded).

Body-weight adjusted levodopa intake (mg levodopa/kg body weight) of patients showed trends of a correlation with the change in performance accuracy across sessions (r = 0.52, p = 0.081), suggesting a larger degradation of performance accuracy in patients with stronger medication, as well as the change in response-related pupil dilation (r = −0.528, p = 0.078), suggesting a more positive change in response-related pupil dilation in patients with lower medication levels.

Most patients (N = 7) received a combined medication of levodopa and a DOPA decarboxylase inhibitor, three patients received only levodopa, and two received levodopa, a DOPA decarboxylase inhibitor and a dopamine agonist. While a statistical comparison of these subgroups is not possible, the ON–OFF effects (i.e., difference between Session 1 and Session 2) for experimental measures showing Group x Session interactions were inspected visually for apparent deviations or outliers. This did not indicate systematic effects of medication type.

## Discussion

The present study investigated the effects of *GCH1* mutations and levodopa medication on time estimation and the associated pupil-linked arousal responses during task and feedback processing. Relative to HC, DRD patients showed comparable time estimation accuracy ON medication but degraded performance OFF medication. In contrast, task-evoked pupil responses showed the opposite pattern. Behavioral trial-to-trial adjustments were smaller in DRD compared to HC, particularly OFF medication. As the order of medication conditions (ON /OFF) was not counterbalanced between DRD patients, the effects are consistent with two alternative explanations, indicating either medication-related alterations in task performance and arousal dynamics or a more general learning deficit in DRD. These are discussed in more details below.

Performance accuracy of HC improved from Session 1 to Session 2, indicating experience-dependent improvement. In contrast, DRD patients showed comparable performance to HC when ON medication but worse performance OFF medication. Acceleration or slowing of the internal clock under dopaminergic medication^13^ was not evident, which is in line with a recent study on D1 receptors in rats^16^. Instead, less dopaminergic influence from the VTA to cortical neurons and from the substantia nigra to MSNs^10^ may lead to impairments in oscillatory and weighting functions OFF medication, while they are normalized ON medication. In line with this reasoning, explorative correlation analyses suggested that higher medication levels were associated with a more pronounced increase in time estimation errors from ON to OFF medication, corroborating the close connection between dopaminergic functioning and task performance.

**Figure 5:**
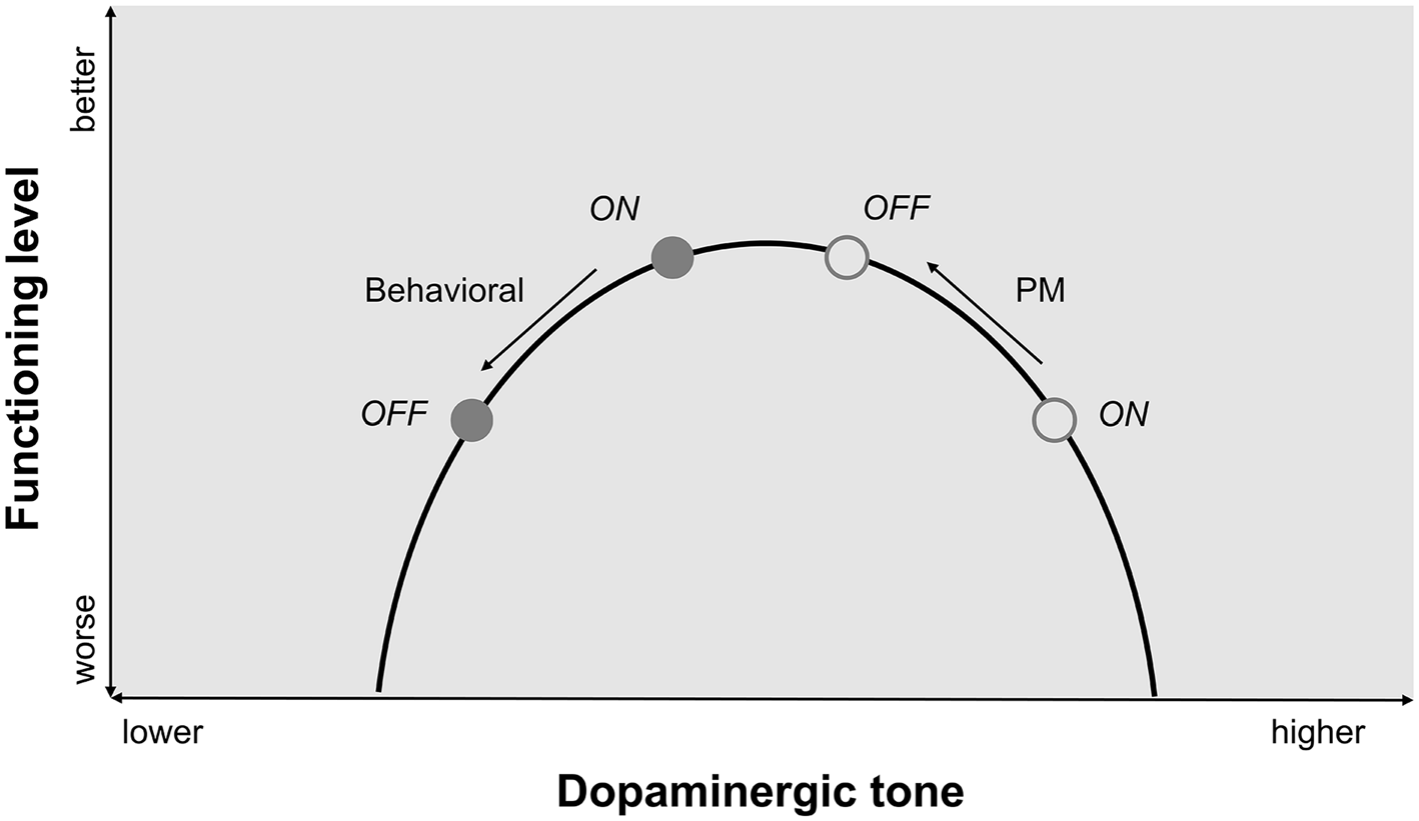
*Relation between dopaminergic tone and functioning.* U-shaped curve for the proposed relation between dopaminergic tone and behavioral and pupillometric (PM) functioning. While behavioral performance degrades in OFF state, pupil responses normalize in OFF state compared to healthy controls^45,46^.

As confirmed by negative association between clinical scores and performance found in patients both ON and OFF levodopa, the time estimation task has a motor component. Also, as to be expected, two of the clinical scores (MDS-UPDRS-III, BFMDRS) showed an increase upon medication withdrawal, raising the possibility that patients might show degraded time estimation accuracy merely as a consequence of more severe motor symptoms when OFF levodopa. However, this explanation seems unlikely as the correlation between differences scores (OFF minus ON) of clinical and experimental measures did not indicate significant associations. Hence, motor functioning and performance in DRD patients could be at or near the peak of the inverted U-shaped curve relating dopaminergic tone to task performance in the ON state and in an upwards slope state OFF medication (Fig. 5)^45,46^.

Dopamine plays an important role in different types of learning^30–34^, and IPD patients show difficulties in learning^33,47^. Furthermore, less activity at D1-receptors in fronto-parietal regions can lead to problems in working memory^48^. Besides that, *GCH1* mutations may cause a general learning deficit in DRD patients, independent of the current dopaminergic deficit. This provides a theoretical alternative explanation for the observed behavioral results. Due to the confound of medication status and session order, effects of learning and medication cannot be discriminated with certainty in our experimental design. However, DRD patients were under medication in the first session, presumably having normalized or even supraphysiological dopamine levels, which speaks against a learning deficit due to dopamine deficiency in that particular session. We therefore consider it more likely that the performance degradation of DRD patients relative to HC from the first (ON) to the second (OFF) session is explained by a dopamine deficiency in the second session.

Performance of our time estimation task also involves processing of feedback. Our analysis indicated reduced trial-to-trial adjustment in DRD patients, particularly, but not exclusively, when OFF levodopa. In case of a distortion of the clock stage in the Scalar Expectancy Model, feedback would lead to correction of time estimation^49^, while changes in the memory stage are stated to be not responsive to feedback^49,50^. The memory stage has been attributed to the cholinergic system^50^, but a dopaminergic influence on working memory is also evident^30^. Therefore, reduced behavioral adjustments could indicate changes in DRD patients beyond simple motor performance, especially when OFF levodopa.

Across both groups and sessions, we found a pronounced task-related pupil dilation. This dilation response is likely driven by processing related to time estimation—specifically, the associated decision processing and action preparation, which occur throughout the interval between the trial onset cue and participant’s motor response^21,44^. Relative to HC, DRD patients showed reduced task-related pupil dilation ON medication and comparable dilation OFF medication. Learning processes initially lead to greater pupil dilation and reduced diameters when the task is being learned^51^. This was the case for healthy controls but not for patients. This could be due to biochemical changes in the noradrenergic system, reduced motivation in ON state or alterations in the dopaminergic system in DRD patients.

A noradrenergic influence on pupil-linked arousal systems is indicated by less noradrenaline in the blood and a smaller amount of 3-methoxy-4-hydroxyphenylglycol in the cerebrospinal fluid of unmedicated DRD patients^17,18^. A supplementation of dopamine could lead to a stabilization, or an excess of noradrenaline concentrations given that dopamine is the precursor for noradrenalin. In our case, changes in noradrenergic innervation could lead to changes in the arousal system of DRD patients. Yet, reliable data about noradrenergic concentrations in brains of DRD patients is missing, so a direct influence of noradrenaline remains speculative.

Given the behavioral results, an overall effect of motivation on pupil linked arousal^19^ in patients is unlikely. Had patients been less motivated in the first session, this should have led to reduced behavioral performance.

Another possible explanation for the pupil results is related to the dopaminergic system. Dopaminergic pathways linked to the VTA^21^ may be overdosed by the supra-physiological levodopa intake, leading to chronically high dopaminergic tone and a blunted dopaminergic contribution to phasic pupil responses. In OFF state, these evoked responses in DRD patients may be normalized due to the pausing of medication (Fig. 5). This view is supported by the trend for a negative association between body weight adjusted levodopa intake and change of pupil response between the sessions. More levodopa intake could lead to a distorted normalization of task-related pupil responses due to the higher amount of stored levodopa.

Arousal responses elicited by feedback processing showed significant task-related effects across groups and sessions^23^. However, feedback-evoked arousal responses did not reveal any group- or session-specific effects. Future work using larger patient samples, manipulations eliciting larger prediction errors, and/or well-controlled monitoring of pupil dynamics and task performance during behavioral training, could shed light on this issue. Specifically, the small number of patients and the fixed order of ON and OFF sessions in patients are limitations of this study resulting from the fact that DRD is a very rare disease and logistical constraints.

To summarize, our results reveal partially levodopa-dependent changes of time estimation, trial-to-trial adjustment, and task-related engagement of pupil-linked arousal in patients with DRD. Our findings suggest that these dopamine-modulated processes have different optimum values of dopamine functioning, while an alteration of noradrenergic function remains speculative. Daily levodopa dosages in these patients are currently predominantly titrated as a function of motor symptoms. To best identify suitable doses for optimally adapted behavior, behavioral tasks focusing on timed motor responses might be helpful to tailor individualized treatment.

## Data Availability

The datasets generated during and analyzed during the current study are available from the corresponding author on reasonable request.
